# Randomized Trial on the Effects of a Mindfulness Intervention on Temperament, Anxiety, and Depression: A Multi-Arm Psychometric Study

**DOI:** 10.3390/bs12030074

**Published:** 2022-03-10

**Authors:** Andrea Poli, Angelo Giovanni Icro Maremmani, Angelo Gemignani, Mario Miccoli

**Affiliations:** 1 Department of Clinical and Experimental Medicine, University of Pisa, 56126 Pisa, Italy; mario.miccoli2020@virgilio.it; 2 Department of Psychiatry, North-Western Tuscany Region NHS Local Health Unit, 55049 Viareggio, Italy; angelo.maremmani@uslnordovest.toscana.it; 3 Association for the Application of Neuroscientific Knowledge to Social Aims (AU-CNS), Pietrasanta, 55045 Lucca, Italy; 4 G. De Lisio Institute of Behavioral Sciences, 56100 Pisa, Italy; 5 Department of Surgical, Medical and Molecular Pathology and of Critical Care Medicine, University of Pisa, 56126 Pisa, Italy; angelo.gemignani@unipi.it

**Keywords:** mindfulness, childhood, temperament, attention, anxiety, depression

## Abstract

Mindfulness is a mental state that can be achieved through meditation. So far, studies have shown that practicing mindfulness on a consistent and regular basis can improve attentional functions and emotional well-being. Mindfulness has recently begun to be used in the field of child development. The goal of this study is to assess if a mindfulness program may help primary school students in reducing anxiety and depression while also improving their temperamental characteristics. This multi-arm pre-post study included 41 subjects recruited in the fifth year of two primary school classes. Participants were randomly assigned to the experimental and control groups. The experimental group, but not the control group, underwent an eight-week mindfulness training. Every week, the program included 60-min group sessions. QUIT (Italian Questionnaires of Temperament) and TAD (Test for Anxiety and Depression in Childhood and Adolescence) were used to assess temperament, and anxiety and depression, respectively. Both groups were administered both instruments before and after mindfulness intervention. The mindfulness program lowered anxiety levels and was effective in changing temperament dimensions: there was an increase in social orientation (SO), positive emotionality (PE), and attention (AT), as well as a decrease in inhibition to novelty (IN) and negative emotionality. Path analysis revealed that AT may promote the improvement of both SO and IN. Similarly, PE may be promoted by the decrease of IN. Clinical implications are discussed.

## 1. Introduction

Mindfulness-based interventions are mainly complex programs with more components cultivating mindfulness, such as breathing awareness practice, working with thoughts and emotions, awareness practices of senses and daily life, kindness practice, etc., and they represent the central teaching of Buddhist practice [1]. In the last twenty years, mindfulness-based interventions have been increasingly representing key elements related to the dialogue between the eastern and the western world. According to Kabat-Zinn, mindfulness could be described as the process of paying particular attention, on purpose, to the present moment, and in a non-judgmental manner [1]. It is conceptualized by modern psychology as the development of a particular type of attention given to the present moment, characterized by acceptance of experience and suspension of judgment, which allows the practitioner to respond in the most appropriate way to any given situation rather than simply reacting to it. Though meditation is not the only way to practice mindfulness, most authors agree that the development of mindfulness is associated with the practice of meditation [2,3,4,5,6,7,8]. The construct of mindfulness is currently used with four different but interconnected meanings: mindfulness as a traditional meditation practice, as a modern clinical intervention, as a state, and as a trait [2]. One of the essential objectives of any psychology or institution that intervenes towards subjects in developmental age is to favor the development and maintenance of the individual’s health and the prevention of physical and mental discomfort [9]. In recent decades, mindfulness-based interventions have been proved to improve mental health and well-being among adults and youth as well [2,5].

The World Health Organization [10] has provided a definition of the concept of health that includes four domains: a state of physical, mental, social, and spiritual well-being. In this perspective, in the general population it has been shown that mindfulness is related to lower levels of depression and anxiety [11]. The estimated number of hours of mindfulness meditation practice did not affect depression or anxiety directly but did reduce these indirectly by increasing mindfulness. Accordingly, emotional regulation plays a significant mediating role between mindfulness and symptoms of depression and anxiety and suggest that meditation focusing on reducing worry and rumination may be especially useful in reducing the risk of developing clinical depression [11]. Meta-analytic evidence also showed that mindfulness-based therapy is a promising intervention for treating anxiety and mood problems in clinical populations [12]. The practice allows the individual to explore their own body dimension in an autonomous, spontaneous, decentralized way and to understand over time the relationships between the cognitive, emotional and physical-sensorial spheres [13,14,15]. This aspect is even more relevant in developmental age, as children often use the body as a metaphor or vehicle of information for their own internal experience. Their body and mind are in constant evolution, it is important for this to favor the development of educational tools and methods aimed at the continuous and progressive integration and harmonization of these two systems [16,17]. The significant role of mental health problems in developmental age and the associated risks underline the importance and the need to evaluate the effectiveness of school prevention programs that aim to promote protective factors and the ability to cope effectively with stress in all children and adolescents. Many school prevention programs have limitations [18], such as focusing on a single development domain, or they are short-term interventions and cannot be integrated and extended within the school curriculum [19,20,21,22,23,24,25,26,27,28,29,30,31]. Current research highlights the usefulness of mindfulness interventions in various problems of developmental age, however to date we have a small amount of controlled and randomized efficacy studies and it is considered useful to further develop research relating to the effects of particular processes developed by mindfulness meditation [32,33]. Hence, this study aims to investigate whether a mindfulness program with primary school children can be effective in reducing anxiety and depression levels and in changing temperament dimensions [30,31,33,34].

Recent research suggests that maternal mindfulness during pregnancy may have positive effects on temperament and infant development. Specifically, this association may be mediated by reduced anxiety symptoms in pregnant women who score high on mindfulness [35]. However, the specific effects of a structured mindfulness intervention on temperamental dimensions of primary school children are not known and we set out to explore the possible effects of mindfulness on children’s temperament. In particular, we planned that, in parallel, an experimental group underwent a mindfulness intervention and a control group underwent daily school activities. Measures related to temperament, anxiety, and depression were administered before and after the intervention. The following were hypothesized: with respect to before intervention measures, the experimental group, but not the control group, would show improvement related to temperament (in particular, in relation to its attentional dimension), anxiety, and depression.

## 2. Materials and Methods

### 2.1. Trial Design

As stated in the ClinicalTrials.gov Identifier NCT05179096 (https://clinicaltrials.gov/ct2/show/NCT05179096; accessed on 2 March 2022; the “Mind the Child study”), our study is an interventional controlled trial with a randomized allocation. The intervention model follows a parallel assignment, and the primary purpose is supportive care. The study was conducted in accordance with the Declaration of Helsinki.

### 2.2. Participants

Eligibility criteria were as follows: ages eligible for study were from 9 to 11 years old, sexes eligible for study were both females and males accepted as healthy volunteers. Inclusion criteria were as follows: children aged 9 to 11 years; children have reasonable comprehension of spoken language and can follow simple instructions; children and their parents are willing to attend all intervention sessions; children and parents have an adequate understanding of Italian. Exclusion criteria: concurrent enrollment in other intervention trials; child or parent regularly practice complementary health interventions such as meditation. The mindfulness program was carried out in an Italian public primary school in Pisa. Parents of all the subjects enrolled in the study provided their written informed consent.

### 2.3. Interventions

Following TIDieR (Template for Intervention Description and Replication) criteria [36], the “Mind the Child study” consisted of 2 arms: an experimental group, undergoing a mindfulness intervention program, and a control/no intervention group, following daily routine school activities. The mindfulness program was carried out by an experienced psychotherapist, certificated as a mindfulness instructor. The experimental group underwent the eight-week mindfulness program consisting of group sessions of 60 min per week [37]. The mindfulness program was originally conceived and conducted according to the protocols described in the literature [30,31] and is described in detail in Supplementary Table S1 [38,39]. Briefly, the intervention was related to mindful eating practice, mindfulness exercises as “paying attention to here and now”, breath-based practices, body scan exercises, and walk practice.

### 2.4. Outcome Measures

#### 2.4.1. Primary Outcome Measures

*Italian Questionnaires of Temperament (QUIT; [40]).* QUIT is a questionnaire consisting of 54 items that measure temperamental dimensions: inhibition to novelty (IN), attention (AT), motor activity (MA), social orientation (SO), positive and negative emotionality (PE and NE, respectively). Each item ranks on a 6-point scale with responses ranging from “rarely” to “often”. The Cronbach α of each of the QUIT dimensions was found to be >0.75 [40]. During this study, the questionnaire was filled in by the teachers. In the current study, all the subscales showed good internal consistency (α’s between 0.76 and 0.85).

#### 2.4.2. Secondary Outcome Measures

*Test for Anxiety and Depression in Childhood and Adolescence (TAD; [41]; Italian version in [42]).* In order to measure anxiety and depression, the TAD (Scale A), a self-report questionnaire filled in by the students, was used [42]. The scale assesses the frequency and severity of symptoms. TAD is comprised of 22 items: 11 items are related to depression, while the other 11 items assess anxious symptoms. TAD is a 4-point, Likert-type, scale which indicates the frequency and severity of each item, with responses ranging from “never” to “often”. Cronbach’s α of the TAD-Scale A is 0.84 [42]. In this study, the questionnaire is filled in by the children. In the current study, this scale showed good internal consistency (α = 0.88). Overall, the questionnaires took between 10 and 25 min to be completed. The scales were administered in a counterbalanced fashion to control for order and sequence effects. No external incentives were offered for participating in this study.

### 2.5. Sample Size

Following recommendations to carry out pilot investigations, a minimum sample size of 12 per group was considered [43] in order to carry out our pilot trial [44,45]. Eighty-nine subjects were assessed for eligibility among public primary school students in Pisa. Among students assessed for eligibility, 43 declined to participate to the intervention, while 5 were not enrolled since children and their parents were not willing to attend all intervention sessions. A total of 41 subjects were enrolled in the study for randomized allocation.

### 2.6. Randomization

Subjects were randomly assigned in a 1:1 ratio to either the experimental intervention group (mindfulness) or the no-intervention group (daily routine school activities) using a computer-generated basic randomization sequence. Randomization was carried out after the baseline assessment by a statistician who was not otherwise involved in the study and had no interaction with the study participants. The allocation was and will be blinded to the outcome evaluators, and participants were told not to reveal their group assignment to the outcome evaluators. The psychologists that carried out the intervention were not the same as the outcome evaluators.

### 2.7. Statistical Analysis

All basic statistical analyzes were performed with SPSS^®^ 27 (IBM Corp., Armonk, NY, USA) and SigmaPlot^®^ 14 (Systat software, Chicago, IL, USA). Shapiro–Wilk test was performed to verify the non-normality of the distributions. In order to compare age between control and mindfulness samples we applied a Mann–Whitney Rank Sum Test (MWRST), while to compare gender frequency we applied the Fisher Exact Test, because over 20% of the expected values in the contingency table were less than five. For comparisons between groups, before treatment, a Friedman’s two-way analysis of variance on ranks (F-tw-ANOVA) with Dunnett post-hoc group rank sums comparisons against a control group were used; while, for comparisons within groups, after treatment, a F-tw-ANOVA with Tukey post-hoc rank sums comparisons were used. In order to identify the best models predicting each of the QUIT subscales, the General Linear Model (GLM) regression analysis was used and all the QUIT variables that showed a significant effect after mindfulness intervention were considered. For each of the QUIT subscales to be predicted as a criterion, the model showing the highest adjusted R^2^ was considered.

In order to explore and confirm a possible path model, obtain goodness-of-fit indices, and maximum-likelihood estimates of model parameters, AMOS 27.0 was employed. For each predictor, the Variance Inflation Factor (VIF) was calculated, and it always fell within the range (1.05–2.64), indicating that there was no significant multicollinearity [46]. The *p* values reported were two-tailed, and a *p* value < 0.05 was considered significant. Before performing path analysis, we analyzed the relationships between the variables. The absolute fit indices utilized in this study were χ^2^ and the root mean square error of approximation (RMSEA); the incremental fit indices used in this investigation were the comparative fit index (CFI) and the Tucker–Lewis index (TLI). CFI and TLI values of 0.90 or higher, and RMSEA values of 0.06 or lower, were considered a “good fit.” A better fit is indicated by χ^2^ values that are closer to zero. Since χ^2^ is sensitive to the sample size employed in the model fit analysis, it was not suggested as a model fit judgement [47]. Hence, it was just reported in this study but not used as a fit statistic. We employed the following model fit criteria [48]: TLI and CFI: ≥0.90 indicated acceptable fit, ≥0.95 indicated excellent fit; RMSEA: ≤0.08 indicated acceptable fit, ≤0.06 indicated excellent fit, and its 90% confidence interval (CI) was reported.

## 3. Results

### 3.1. Group Comparisons

**Figure 1:**
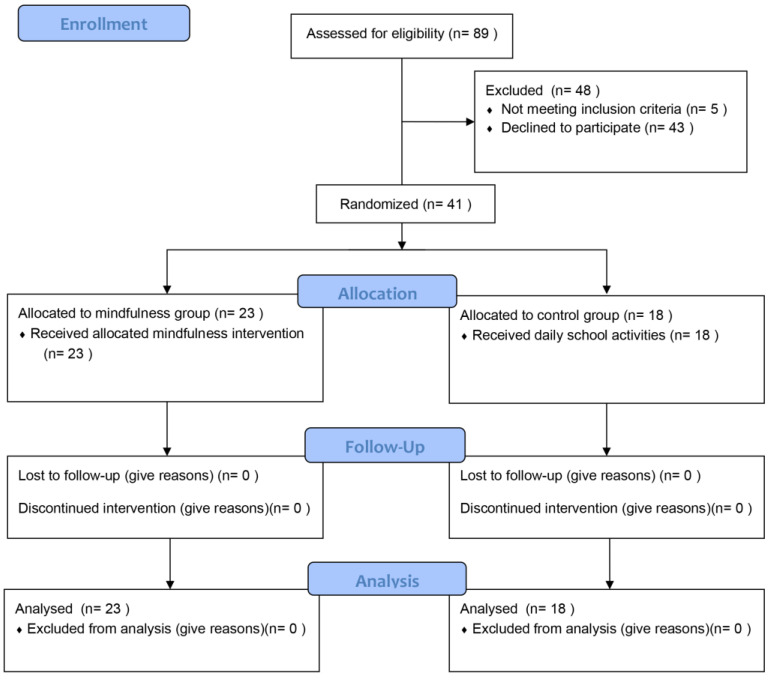
CONSORT flow diagram of the study.

As reported in the CONSORT flow diagram [49] of the study in Figure 1, 41 subjects (34.15% females; mean age = 10.78, SD = 0.38) belonging to two fifth-year primary school classes were included in the study. The control group was composed by 18 subjects (4 females, 22.22%) and the mindfulness group was composed by 23 subjects (10 females, 43.48%). As a first step, we compared gender frequency and age between control and mindfulness samples to evaluate gender and age homogeneity among groups. Fisher Exact Test revealed that gender frequency was not significantly different (*p* = 1), between the two groups, while MWRST showed that age was not significantly different (*p* = 0.874), as well. Thus, control and mindfulness groups were homogeneous regarding gender and age. Hence, we compared the scores shown by participants between the two groups, before mindfulness intervention, and within the two groups, after mindfulness intervention, regarding scales and subscales of the study measures. Regarding pre-intervention baseline measures, Dunnett’s post-hoc analysis of F-tw-ANOVA revealed that control and mindfulness samples did not show scores that were significantly different for the TAD (TAD-Anxiety: *p* = 0.378; TAD-Depression: *p* = 0.271), and the QUIT (QUIT-SO: *p* = 0.738; QUIT-IN: *p* = 0.198; QUIT-MA: *p* = 0.207; QUIT-PE: *p* = 0.132; QUIT-NE: *p* = 0.792; QUIT-AT: *p* = 0.566). Conversely, comparing post-intervention measures, Dunnett’s post-hoc analysis of F-tw-ANOVA revealed that control and mindfulness samples did not show a significant difference regarding TAD (TAD-Anxiety: *p* = 0.269; TAD-Depression: *p* = 0.386) but, with respect to control sample, mindfulness sample showed a significant increase in SO (QUIT-SO: *p* = 0.002), MA (QUIT-MA: *p* = 0.005), PE (QUIT-PE: *p* < 0.001) and AT (QUIT-AT: *p* < 0.001) subscales, as well as a decrease in IN (QUIT-IN: *p* < 0.001) and in NE (QUIT-NE: *p* = 0.004) subscales (Table 1).

In order to identify the effects of mindfulness intervention on each group, we compared separately control and mindfulness groups before and after mindfulness intervention. As expected, Tukey’s post-hoc test of F-tw-ANOVA revealed that the control sample, after the mindfulness intervention, did not show scores that were significantly different for the TAD (TAD-Anxiety: *p* = 0.602; TAD-Depression: *p* = 0.775), and the QUIT (QUIT-SO: *p* = 0.908; QUIT-IN: *p* = 0.694; QUIT-MA: *p* = 0.991; QUIT-PE: *p* = 0.954; QUIT-NE: *p* = 0.881; QUIT-AT: *p* = 0.871). Conversely, Tukey’s post-hoc test of F-tw-ANOVA revealed that the mindfulness sample, after the mindfulness intervention, showed a significant improvement regarding anxiety (TAD-Anxiety: *p* = 0.006), but not in depression symptoms (TAD-Depression: *p* = 0.756). Regarding QUIT dimensions, the mindfulness sample, after the mindfulness intervention, showed a significant increase in SO (QUIT-SO: *p* < 0.001), PE (QUIT-PE: *p* < 0.001) and AT (QUIT-AT: *p* < 0.001) subscales, as well as an improvement in IN (QUIT-IN: *p* < 0.001) and in NE (QUIT-NE: *p* < 0.001) subscales (Table 2).

### 3.2. GLM Regressions

In order to identify the best models predicting each of the QUIT subscales, we carried out the best GLM regression analysis and all the QUIT variables that showed a significant effect after mindfulness intervention were considered (with the exception of MA). The VIF was computed for each predictor and for all significant predictors always fell within the range (1.16–2.54) which is considered as evidence of a lack of substantial multicollinearity [50]. Results of the best subset regression analysis predicting each of the significant QUIT subscales for mindfulness group are reported in Table 3.

First, we evaluated which of the QUIT variables were able to predict QUIT-SO. QUIT-AT subscale was a unique significant predictor (β = 0.571, *p* = 0.015) of QUIT-SO subscale. When inspecting which of the QUIT variables were able to predict QUIT-IN, QUIT-PE, and QUIT-AT were found to be significant predictors (β = −0.536, *p* = 0.022 for QUIT-PE; β = 0.394, *p* = 0.04 for QUIT-AT). QUIT-PE was found to be uniquely predicted by QUIT-IN (β = −0.482, *p* = 0.022), while QUIT-AT was found to be predicted by both QUIT-SO and QUIT-IN (β = 0.506, *p* = 0.015 for QUIT-SO; β = −0.543, *p* = 0.04 for QUIT-IN). QUIT-NE was not found to be predicted by any of the QUIT subscales.

### 3.3. Path Analysis

Path analytic models were tested using AMOS 27 for mindfulness group post-intervention to evaluate if there was a possible particular association among QUIT subscales. Skewness ranged from −1.21 to 0.87, and kurtosis ranged from −1.1 to 0.91. When using path analysis or structural equation modeling (SEM), acceptable skewness values range from −3 to +3, and acceptable kurtosis values range from −10 to +10 [45,46]. Values that are below or above these ranges are questionable, although path analysis and SEM are highly robust analytical methods, thus minor deviations may not indicate severe assumptions violations [51]. We used the Maximum Likelihood (ML) estimator.

**Figure 2:**
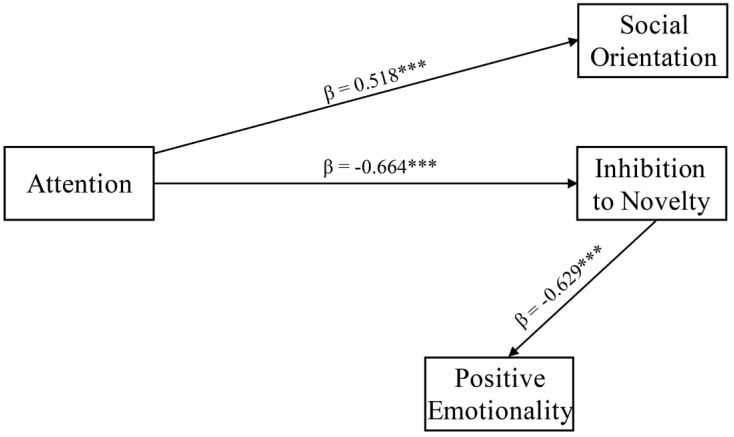
Path analytic model related to QUIT subscales for Mindfulness sample post-intervention (*n* = 23). *** indicates *p* < 0.001.

In the mindfulness group post-intervention, among QUIT subscales, we found that AT was able to significantly predict both SO (β = 0.518, *p* < 0.001, SE = 0.134) and IN (β = −0.664, *p* < 0.001, SE = 0.197). Furthermore, PE was found to be significantly predicted by IN (β = −0.629, *p* < 0.001, SE = 0.163). Overall, AT was found to be the unique QUIT subscale to be able to predict both SO and IN (Figure 2). In turn, IN was found to be the only QUIT subscale that was able to predict PE (χ^2^ (2) = 0.186, *p* = 0.911; CFI = 0.996, TLI = 0.98, RMSEA = 0.024 [0.021; 0.027]).

## 4. Discussion

Our study aimed at evaluating the effects of a mindfulness intervention on temperament, anxiety, and depression of primary school children (aged 9–11), with respect to children undergoing daily school activities. Our results demonstrate that the mindfulness program was able to significantly reduce anxiety levels [34], but not depressive symptomatology [52]. In addition, mindfulness intervention was able to improve the temperamental dimensions of AT, SO, PE, IN, and NE.

A recent study found that mindfulness interventions in adolescents not only was able to reduce anxiety, but also changed the amygdala’s network features, enhancing structural connectivity [53,54]. In addition, it has been shown that mindfulness-based interventions are effective programs for treating anxiety and mood problems in adult clinical populations [12], as well as for reducing the risk of developing clinical depression through the reduction in worry and rumination [11]. However, we found that the mindfulness program was able to significantly reduce anxiety, but not depression, levels in primary school children ranging from 9 to 11 years old. Interestingly, in a recent meta-analysis it has been also highlighted that mindfulness-based interventions can be effective in children and adolescents with mental health symptoms. Remarkably, in non-clinical populations compared to non-active control, mindfulness-based interventions were also effective improving anxiety and stress but not depression, as in our study [55,56]. Since it has been demonstrated that high levels of shame and maladaptive guilt were related to the onset of preschool depression, as early as age 3 [57], and that shame and guilt exert negative impacts on cognition and attention [58], our non-clinical samples, unlike clinical samples, may not show significant levels of shame or guilt related to an actual clinical manifestation of depression and thus may already show healthy mood levels at baseline that may not necessitate an improvement.

The program was found to be effective in changing the temperamental dimensions, there was an increase in the levels of SO, PE, and AT and a reduction in the levels of IN and NE [52,59], with robust effect magnitudes even in a non-clinical sample [12]. The dimension of MA does not show variations in response to the intervention, possibly because the QUIT evaluates its temporal aspect. QUIT evaluates MA as the speed through which the responses are issued and does not consider the energetic and resistance components, that may be detected by using another tool [56]. The mindfulness program was particularly effective at promoting the development of the dimensions of cognitive performance, such as attention [60], and of prosociality, such as positive emotions and SO [52]. Since these are necessary dimensions for monitoring interpersonal conflict [61], it can be hypothesized that, in children, the practice of mindfulness is able to promote, and possibly improve, the attentional and prosocial resources: they have the opportunity to learn to master an accessible technique that they can use to manage their emotion and to calm down. Research has shown that aggressive/rejected children had difficulties in shifting their attention from a negative to a prosocial affect [62]. After experiencing social failure, aggressive/rejected children were found to be less able to properly manage their behavior, confirming that attention has an important role in prosociality and positive emotionality [62]. In addition, while prior research has shown that prosocial attitudes contribute to attentional broadening, other findings demonstrate that attentional broadening leads to increased prosociality as well, implying a reciprocal relationship between attention and prosocial behavior [63]. It could be also hypothesized that reorienting attention could be a very important effect of our mindfulness intervention. In fact, it has been demonstrated that temporo-parietal-junction (TPJ), that plays a role in reorienting attention, has also been implicated in the inference of other’s effort during movement and the cost of helping. Thus, TPJ, together with its role in reorienting attention, may contribute to higher-level social cognition [64].

Considering the effects of mindfulness on attention [65], it can be hypothesized that, after a mindfulness intervention, the temperamental dimension identified by the QUIT can be influenced as proposed in Figure 2. AT dimension, the main target of the mindfulness intervention, may promote the improvement of both SO and IN dimensions. It may also be hypothesized that, as a positive feedback mechanism, SO and IN dimensions may favor the improvement of the AT dimension. Similarly, PE may be promoted by the decrease of the IN dimension that, in turn, may favor the decrease of the IN dimension, as an additional positive feedback mechanism. Accordingly, it has been shown that adolescents who were behaviorally inhibited as toddlers and young children showed heightened attention bias to threat. More importantly, attention bias to threat moderated the relation between childhood behavioral inhibition and adolescent social withdrawal [66]. Furthermore, over time behavioral inhibition in toddlerhood predicted high levels of social withdrawal in early childhood and this relation was moderated by attention bias [67]. Thus, it can be hypothesized that in our non-clinical sample the mindfulness program, exerting its main effects on attention, directly promoted prosociality and proneness to novelty that, in turn, promoted positive emotions.

There is substantial evidence that the COVID-19 pandemic has had a significant psychological impact as a result of the imposed limitations [68,69,70]. Previous research has extensively shown that mindfulness meditation can be used as a protective factor in several populations, like children [70]. Mindfulness can be able to buffer the negative effects of social media exposure on psychological distress [71]. During the pandemic, teachers practicing mindfulness meditation had a positive impact. The effects of mindfulness on affective empathy, emotional tiredness, anxiety, and depression among Italian female teachers demonstrated that contemplative practices improved psychological well-being and interoceptive awareness [72]. Because there is so much information in the literature regarding the excellent impacts of mindfulness on our health and well-being, it is reasonable to assume that this emotional well-being is linked to improved learning. However, learning and practicing mindfulness is a lengthy process that requires both time and space, so future research should concentrate on concerns of sustainability and group variations. It may take some time to learn mindfulness, depending on how engaged and motivated each individual is.

Finally, the following limitations should, however, be considered. (a) Our samples were relatively small, further studies should replicate our findings in larger samples. (b) Participants were self-selected; this might limit the generalizability of our conclusions. (c) We included a single temperament measure in our study, the QUIT. Future research could replicate our results using other temperamental measures. (d) All of the TAD and QUIT data were measured through a single source (self-rating and teacher rating, respectively). The conclusions would be strengthened by the inclusion of both self- and teacher-ratings for both TAD and QUIT measures. (e) Self-report data tends to inflate associations among variables.

## 5. Conclusions

Despite these limitations, our study demonstrated that a mindfulness program in a group of primary school children (9–11 years old), with respect to a matched non-active control group, was able to improve anxiety-related symptoms and the temperamental dimensions of SO, PE, AT, IN, and NE.

Our results promote the implementation of mindfulness interventions for primary school children in order to promote anxiety symptoms prevention. More importantly, mindfulness interventions may represent an effective training for reducing loneliness and increasing social contact in daily life [73] and even for improving cognitive function by increasing the neuronal expression of miRNA-29c [74]. Furthermore, a positive relationship was also found between childhood trauma and absorption and depersonalization, as well as a significant negative association between mindfulness and absorption and depersonalization [75], so mindfulness programs may represent effective interventions to promote integration in clinical [76] and non-clinical individuals [77].

## Figures and Tables

**Table 1:** Group comparisons among the study measures between Mindfulness (*n* = 23) and Control (*n* = 18) samples assessed pre-intervention and post-intervention.

Variable	Control—Pre	Mindfulness—Pre	*p*	Control—Post	Mindfulness—Post	*p*
1. TAD-Anxiety	105 (12.24)	109.5 (13.07)	0.378	99.70 (17.89)	102.75 (13.42)	0.269
	102.5 [35]	115 [40]		90 [65]	100 [45]	
2. TAD-Depression	106.07 (17.34)	105.75 (9.07)	0.271	102.35 (14.15)	103.5 (15.31)	0.386
	105 [65]	102.5 [40]		105 [60]	105 [60]	
3. QUIT-SO	4.37 (0.97)	4.55 (0.84)	0.738	4.35 (0.98)	5.32 (0.61)	0.002
	4.19 [3.5]	4.63 [2.87]		4.15 [3.5]	5.5 [1.87]	
4. QUIT-IN	3.84 (1.27)	3.08 (0.94)	0.198	3.82 (1.25)	2.06 (0.67)	<0.001
	4.11 [4.44]	3.11 [3.78]		4 [4.44]	1.89 [2.56]	
5. QUIT-MA	3.05 (0.95)	3.59 (1.18)	0.207	3.03 (0.93)	3.53 (0.44)	0.004
	2.8 [3.9]	3.4 [3.7]		2.8 [3.9]	3.5 [1.9]	
6. QUIT-PE	2.91 (1.34)	3.61 (0.84)	0.132	2.90 (1.34)	4.86 (0.56)	<0.001
	2.61 [4.34]	3.67 [4]		2.65 [4.4]	5 [1.89]	
7. QUIT-NE	3.61 (1.44)	3.45 (0.94)	0.792	3.59 (1.45)	2.14 (0.68)	0.004
	3.61 [4.44]	3.44 [3.22]		3.6 [4.44]	2.11 [2.44]	
8. QUIT-AT	3.21 (1.11)	3.88 (0.81)	0.566	3.19 (1.13)	4.93 (0.74)	<0.001
	3.11 [3.66]	3.89 [3.44]		3.1 [3.66]	5.11 [2.78]	

Note: *p* = *p*-value resulting from Dunnett’s post-hoc test of F-tw-ANOVA; TAD-Anxiety = Test for Anxiety and Depression in Childhood and Adolescence—Anxiety; TAD-Depression = Test for Anxiety and Depression in Childhood and Adolescence—Depression; QUIT-SO = Italian Questionnaires of Temperament—Social Orientation; QUIT-IN = Italian Questionnaires of Temperament—Inhibition To Novelty; QUIT-MA = Italian Questionnaires of Temperament—Motor Activity; QUIT-PE = Italian Questionnaires of Temperament—Positive Emotionality; QUIT-NE = Italian Questionnaires of Temperament—Negative Emotionality; QUIT-AT = Italian Questionnaires of Temperament—Attention. Mean and standard deviation (in brackets), and median and interquartile range (in square brackets) are shown.

**Table 2:** Group comparisons among the study measures within Mindfulness (*n* = 23) and Control (*n* = 18) samples assessed pre-intervention and post-intervention.

Variable	Control—Pre	Control—Post	*p*	Mindfulness—Pre	Mindfulness—Post	*p*
1. TAD-Anxiety	105 (12.24)	99.70 (17.89)	0.602	109.5 (13.07)	102.75 (13.42)	0.006
	102.5 [35]	90 [65]		115 [40]	100 [45]	
2. TAD-Depression	106.07 (17.34)	102.35 (14.15)	0.775	105.75 (9.07)	103.5 (15.31)	0.756
	105 [65]	105 [60]		102.5 [40]	105 [60]	
3. QUIT-SO	4.37 (0.97)	4.35 (0.98)	0.908	4.55 (0.84)	5.32 (0.61)	<0.001
	4.19 [3.5]	4.15 [3.5]		4.63 [2.87]	5.5 [1.87]	
4. QUIT-IN	3.84 (1.27)	3.82 (1.25)	0.893	3.08 (0.94)	2.06 (0.67)	<0.001
	4.11 [4.44]	4 [4.44]		3.11 [3.78]	1.89 [2.56]	
5. QUIT-MA	3.05 (0.95)	3.03 (0.93)	0.991	3.59 (1.18)	3.53 (0.44)	0.715
	2.8 [3.9]	2.8 [3.9]		3.4 [3.7]	3.5 [1.9]	
6. QUIT-PE	2.91 (1.34)	2.90 (1.34)	0.954	3.61 (0.84)	4.86 (0.56)	<0.001
	2.61 [4.34]	2.65 [4.4]		3.67 [4]	5 [1.89]	
7. QUIT-NE	3.61 (1.44)	3.59 (1.45)	0.881	3.45 (0.94)	2.14 (0.68)	<0.001
	3.61 [4.44]	3.6 [4.44]		3.44 [3.22]	2.11 [2.44]	
8. QUIT-AT	3.21 (1.11)	3.19 (1.13)	0.871	3.88 (0.81)	4.93 (0.74)	<0.001
	3.11 [3.66]	3.1 [3.66]		3.89 [3.44]	5.11 [2.78]	

Note: *p* = *p*-value resulting from Tukey’s post-hoc test of F-tw-ANOVA; TAD-Anxiety = Test for Anxiety and Depression in Childhood and Adolescence—Anxiety; TAD-Depression = Test for Anxiety and Depression in Childhood and Adolescence—Depression; QUIT-SO = Italian Questionnaires of Temperament—Social Orientation; QUIT-IN = Italian Questionnaires of Temperament—Inhibition To Novelty; QUIT-MA = Italian Questionnaires of Temperament—Motor Activity; QUIT-PE = Italian Questionnaires of Temperament—Positive Emotionality; QUIT-NE = Italian Questionnaires of Temperament—Negative Emotionality; QUIT-AT = Italian Questionnaires of Temperament—Attention. Mean and standard deviation (in brackets), and median and interquartile range (in square brackets) are shown.

**Table 3:** GLM regression analyses predicting QUIT-SO, QUIT-IN, QUIT-PE, QUIT-NE and QUIT-AT subscales score from the other QUIT subscales for Mindfulness sample post-intervention (n = 23).

Predictor	β	*t*	*p*
Criterion: QUIT-SO			
Adjusted R^2^ = 0.447			
QUIT-IN	−0.038 (0.294)	−0.132	0.897
QUIT-PE	0.052 (0.309)	0.169	0.868
QUIT-NE	−0.244 (0.206)	1.186	0.251
QUIT-AT	0.571 (0.211)	2.705	0.015
Criterion: QUIT-IN			
Adjusted R^2^ = 0.705			
QUIT-SO	−0.025 (0.189)	−0.132	0.897
QUIT-PE	−0.536 (0.214)	−2.503	0.022
QUIT-NE	0.071 (0.171)	0.418	0.681
QUIT-AT	0.394 (0.178)	−2.213	0.040
Criterion: QUIT-PE			
Adjusted R^2^ = 0.624			
QUIT-SO	0.03 (0.179)	0.169	0.868
QUIT-IN	−0.482 (0.193)	−2.503	0.022
QUIT-NE	−0.273 (0.149)	−1.829	0.084
QUIT-AT	−0.031 (0.190)	−0.166	0.87
Criterion: QUIT-NE			
Adjusted R^2^ = 0.465			
QUIT-SO	0.297 (0.25)	1.186	0.251
QUIT-IN	0.135 (0.322)	0.418	0.681
QUIT-PE	−0.574 (0.314)	−1.829	0.084
QUIT-AT	−0.239 (0.27)	−0.883	0.389
Criterion: QUIT-AT			
Adjusted R^2^ = 0.67			
QUIT-SO	0.506 (0.187)	2.705	0.015
QUIT-IN	−0.543 (0.245)	−2.213	0.04
QUIT-PE	−0.048 (0.291)	−0.166	0.87
QUIT-NE	−0.174 (0.197)	−0.883	0.389

Note: Standard errors in parentheses; QUIT-SO = Italian Questionnaires of Temperament—Social Orientation; QUIT-IN = Italian Questionnaires of Temperament—Inhibition To Novelty; QUIT-PE = Italian Questionnaires of Temperament—Positive Emotionality; QUIT-NE = Italian Questionnaires of Temperament—Negative Emotionality; QUIT-AT = Italian Questionnaires of Temperament—Attention.

## Data Availability

The data presented in this study are available on request from the corresponding author. The data are not publicly available due to privacy issue.

## Supplementary Materials

The following supporting information can be downloaded at: https://www.mdpi.com/article/10.3390/bs12030074/s1, Table S1: Overview of the 8-week Mindfulness-Based Stress Reduction intervention.
