# Supplementary material for: Randomized Trial on the Effects of a Mindfulness Intervention on Temperament, Anxiety, and Depression: A Multi-Arm Psychometric Study
Source: Behav Sci (Basel). 2022 Mar 10;12(3):74. doi: 10.3390/bs12030074 (PMC8945710; doi:10.3390/bs12030074)
Supplement: Supplementary file 1 [file behavsci-12-00074-s001.zip › behavsci-1514716-supplementary.pdf]

Supplementary Materials

*Supplementary Table S1***.** *Overview of the 8-week Mindfulness-Based Stress Reduction intervention*

	**Class Agenda**	**Home Practice**
Introduction to Program (parents only) 2 hours	• Mindful Eating<br>• Review data on benefits of mindfulness for adults and children<br>• Review the rationale for offering MBSR to children<br>• Discuss course structure and time commitment<br>• Answer questions	None
Class 1	• Mindful Eating Practice<br>• Introduce Still Quiet Place<br>• Mindfulness as "paying attention to here and now"<br>• Begin breath-based practices such as Jewel/Treasure	• Jewel/Treasure Exercise<br>• Monitor pleasant experiences using Pleasant Experiences Calendar<br>• Engage in one mindful activity (e.g., brush teeth, shower, do a chore, care for a pet)
Class 2	• Mindful Eating Practice<br>• Review class 1 and home practice Jewel/Treasure Exercise<br>• Answer questions about the practice	• Same as Class 1<br>• Eat a snack or meal mindfully
Class 3	• Mindful Eating Practice<br>• Review class 2 and home practice<br>• Introduce concept of "funny mind" (internal dialogue, see Home Practice section)<br>• Body Scan Exercise (mindfulness of bodily experiences)	• Body Scan Exercise (mindfulness of bodily experiences)<br>• Monitor unpleasant experiences using the Unpleasant Experiences Calendar Notice "funny mind"<br>• Notice times when you feel stressed Engage in mindful activity

Class 4	• Mindful Eating Exercise<br>• Review class 3 and home practice<br>• Exercises to explore perception — how do we view ourselves and each other?<br>• Exercises to investigate thoughts associated with difficult tasks<br>• Yoga	• Body Scan Exercise/Yoga (mindfulness of bodily experiences during yoga)<br>• Monitor unpleasant experiences using the Unpleasant Experiences Calendar<br>• Use awareness of breath to slow things down in everyday life Engage in mindful activity
Class 5	• Mindful Eating Practice<br>• Review class 4 and home practice<br>• Explore thoughts and feelings associated with unpleasant experience<br>• Begin to develop concept of “funny mind”<br>• Feelings Practice	• Continue Feelings Practice, using haiku, other poetry, or art to depict feelings<br>• Notice moments of reactivity and explore ways of responding<br>• Engage in new mindful activity
Class 6	• Mindful Eating Practice<br>• Review previous class topics and home practice<br>• Explore Feelings Practice through haiku, art, etc.<br>• Thought Parade Exercise<br>• Walking Practice<br>• Moving our practice into the world	• Thought Parade Exercise<br>• Take a “Thoreau walk”<br>• Feelings Practice<br>• Difficult Communication Calendar<br>• Continue responding to stressful situations and to “funny mind”
Class 7	• Mindful Eating Exercise<br>• Review class 6 and home practice<br>• Communication dyads (one person describes a difficult communication; the other listens and reflects, then they reverse roles)	• Loving-Kindness Exercise<br>• Continue responding to stressful situations and “funny mind”<br>• New, more challenging mindful activity<br>• Imagine the world from someone

	• Share examples of responding, and role-play new responses to situations when the children reacted<br>• Introduce Loving-Kindness Practice	else's point of view<br>• Bring something to share for the last session that represents what the class has meant to you
Class 8	• Group choice<br>• Review class 7 and home practice<br>• Letter to a friend<br>• Making the practice your own	• Your choice<br>• Flashlight Exercise<br>• Make a commitment as to how you will continue
